# Endogenous sex hormones and prostate cancer: a quantitative review of prospective studies

**DOI:** 10.1038/sj.bjc.6690445

**Published:** 1999-06

**Authors:** N E Eaton, G K Reeves, P N Appleby, T J Key

**Affiliations:** Imperial Cancer Research Fund,Cancer Epidemiology Unit, Gibson Building, Radcliffe Infirmary, Woodstock Road, Oxford, OX2 6HE, UK

**Keywords:** prostate cancer, steroid hormones, androstanediol glucuronide, prospective studies, review

## Abstract

This paper presents a quantitative review of the data from eight prospective epidemiological studies, comparing mean serum concentrations of sex hormones in men who subsequently developed prostate cancer with those in men who remained cancer free. The hormones reviewed have been postulated to be involved in the aetiology of prostate cancer: androgens and their metabolites testosterone (T), non-SHBG-bound testosterone (non-SHBG-bound T), di-hydrotestosterone (DHT), androstanediol glucuronide (A-diol-g), androstenedione (A-dione), dehydroepiandrosterone sulphate (DHEAS), sex hormone binding globulin (SHBG), the oestrogens, oestrone and oestradiol, luteinizing hormone (LH) and prolactin. The ratio of the mean hormone concentration in prostate cancer cases to that of controls (and its 95% confidence interval (CI)) was calculated for each study, and the results summarized by calculating the weighted average of the log ratios. No differences in the average concentrations of the hormones were found between prostate cancer cases and controls, with the possible exception of A-diol-g which exhibited a 5% higher mean serum concentration among cases relative to controls (ratio 1.05, 95% CI 1.00–1.11), based on 644 cases and 1048 controls. These data suggest that there are no large differences in circulating hormones between men who subsequently go on to develop prostate cancer and those who remain free of the disease. Further research is needed to substantiate the small difference found in A-diol-g concentrations between prostate cancer cases and controls. © 1999 Cancer Research Campaign


*British Journal of Cancer* (1999) **80**(7), 930–934
© 1999 Cancer Research Campaign
Article no. bjoc.1999.0445

Review

### Endogenous sex hormones and prostate cancer: a quantitative review of prospective studies

**NE Eaton, GK Reeves, PN Appleby and TJ Key**

Imperial Cancer Research Fund, Cancer Epidemiology Unit, Gibson Building, Radcliffe Infirmary, Woodstock Road, Oxford OX2 6HE, UK

**Summary** This paper presents a quantitative review of the data from eight prospective epidemiological studies, comparing mean serum concentrations of sex hormones in men who subsequently developed prostate cancer with those in men who remained cancer free. The hormones reviewed have been postulated to be involved in the aetiology of prostate cancer: androgens and their metabolites testosterone (T), non-SHBG-bound testosterone (non-SHBG-bound T), di-hydrotestosterone (DHT), androstanediol glucuronide (A-diol-g), androstenedione (A-dione), dehydroepiandrosterone sulphate (DHEAS), sex hormone binding globulin (SHBG), the oestrogens, oestrone and oestradiol, luteinizing hormone (LH) and prolactin. The ratio of the mean hormone concentration in prostate cancer cases to that of controls (and its 95% confidence interval (CI)) was calculated for each study, and the results summarized by calculating the weighted average of the log ratios. No differences in the average concentrations of the hormones were found between prostate cancer cases and controls, with the possible exception of A-diol-g which exhibited a 5% higher mean serum concentration among cases relative to controls (ratio 1.05, 95% CI 1.00–1.11), based on 644 cases and 1048 controls. These data suggest that there are no large differences in circulating hormones between men who subsequently go on to develop prostate cancer and those who remain free of the disease. Further research is needed to substantiate the small difference found in A-diol-g concentrations between prostate cancer cases and controls.

**Keywords:** prostate cancer; steroid hormones; androstanediol glucuronide; prospective studies; review

The incidence of prostate cancer varies widely, with Western countries having rates 30–50 times higher than those in Far-Eastern countries (Parkin et al, 1992) and it is now the commonest incident cancer and the second most common cause of cancer mortality in North American men (Parker et al, 1996). The reasons for the wide differences in prostate cancer incidence between populations are not yet established, but the fact that an increase in prostate cancer has been seen among Asian migrants to the USA (Shimizu et al, 1991) suggests that environmental or lifestyle factors are important. However, other than race, age and a family history of prostate cancer (Nomura and Kolonel, 1991), aetiological risk factors remain largely unknown, although it has been suggested that the adoption of Western dietary habits with a high fat and/or low plant food consumption may be associated with prostate cancer risk (Adlercreutz, 1990). Furthermore, it has been postulated that dietary factors may exert their effects by altering sex hormone metabolism, and that this may play a significant role in the progression of latent lesions into clinically relevant cancer (Montie and Pienta, 1994).

Many epidemiological studies have been conducted over the last 20 years in an attempt to identify differences in sex hormone metabolism between prostate cancer patients and controls, and in individuals from high- and low-risk populations, by measuring differences in serum hormone levels. The evidence from case-control studies that circulating hormone levels are related to prostate cancer is inconsistent (Flanders, 1986), and these studies suffer from the problem that disease status may well alter serum concentrations of androgens. As such, it is impossible to interpret any differences present in serum levels between cases and controls in relation to the aetiology of the disease. Prospective studies reduce the possibility that changes in hormone levels are due to disease status as blood samples are taken some years prior to diagnosis. This paper presents a quantitative review (meta-analysis) of the published data from prospective studies which compared serum concentrations of sex hormones in men who subsequently developed prostate cancer with those in men who remained cancer free.

*Received 25 November 1998*
*Accepted 4 January 1999*

*Correspondence to:* NE Eaton

### MATERIALS AND METHODS

Papers were identified by searching the Medline database, 1966–1998, using combinations of the following keywords: hormones; androgens; prostate cancer; prospective; serum. The reference lists of the relevant papers were also examined. Ten papers reporting results from eight prospective studies were identified for inclusion in this meta-analysis, of which seven were nested case-control studies where controls were chosen according to specific matching criteria (Nomura et al, 1988, 1996; Comstock et al, 1993; Hsing and Comstock, 1993; Carter et al, 1995; Gann et al, 1996; Guess et al, 1997; Vatten et al, 1997; Dorgan et al, 1998) and one was a cohort study in which the controls were all participants with no diagnosis of prostate cancer (Barrett-Connor et al, 1990). Two papers (Nomura et al, 1988; Comstock et al, 1993) include cases that are a subset of the prostate cancer cases described in other papers (Nomura et al, 1996; Hsing and Comstock, 1993), but are included in this review as they have analysed different hormones.

Table 1 shows the characteristics of the study populations. All studies, except Barrett-Connor et al (1990), included matching for

**Table 1** Characteristics of the study populations and follow-up times for the seven nested case-control studies and one prospective cohort study

| Reference | Study population | Study period (recruitment to end of follow-up) | Age at recruitment (years) | Mean years to diagnosis (range) | No. cases | No. controls | Matching criteria |
|---|---|---|---|---|---|---|---|
| Nomura et al (1988, 1996) | 6860 participants of the Honolulu Heart Program, Oahu, Hawaii | 1971–1996[a] | 45–68 | 7 (< 1–14)[b] | 141[c] | 141[c] | Age<br>Ethnicity (Japanese-American)<br>Date of venipuncture<br>Time of day of venipuncture<br>No prior history of prostatic surgery |
| Barrett-Connor et al (1990) | 1008 participants of the Rancho Bernardo Study, CA, USA | 1972–1986 | 40–79 | 8 (1–14) | 57 | 951 | Time of day of venipuncture |
| Hsing & Comstock (1993) and Comstock et al (1993) | 25 620 residents of Washington County, MD, USA | 1974–1987 | 35–94 | Not given (1–12) | 98[d] | 98[d] | Age<br>Ethnicity (Caucasian) |
| Carter et al (1995) | 1459 participants of the Baltimore Longitudinal Study of Aging, Baltimore, MD, USA | 1958–1990 | 55–90 | Not given (7–25) | 16 | 16 | Age<br>Date of venipuncture |
| Gann et al (1996) | 22 071 participants of the Physicians' Health Study, USA | 1982–1992 | 40–84 | 6 (range not given) | 222 | 390 | Age<br>Date of venipuncture<br>No prior history of prostatic surgery<br>Smoking |
| Guess et al (1997) | 128 992 participants of the Kaiser Permanente Medical Care Program (KPMCP), US | 1964–1987 | Not given | 14 (5–23) | 106 | 106 | Age<br>Ethnicity (Caucasian)<br>Date of venipuncture<br>Clinic location |
| Vatten et al (1997) | 28 000 blood donors, Oslo, Norway | 1973–1994 | 42–66 | 10 (1–19) | 59 | 180 | Age<br>Date of venipuncture |
| Dorgan et al (1998) | 29 133 participants of the Alpha-Tocopherol, Beta-Carotene Study (ATBC), Finland | 1985–1993 | 50–69 | 4[e] (< 1–7) | 116 | 231 | Age<br>Ethnicity (Caucasian)<br>Date of venipuncture<br>Time of day of venipuncture<br>Smoking (all smokers)<br>Clinic location |

[a]Nomura et al (1988) follow-up period: 1971–1984. [b]Not given in Nomura et al (1988) paper. [c]Nomura et al (1988) included 98 matched case-control pairs which are a subset of Nomura et al (1996) study population. [d]Comstock et al (1993) included 81 matched case-control pairs which are a subset of Hsing et al (1993) study population. [e]Median given.

age. Most populations were predominantly Caucasian, although in one study the participants were all Japanese-American (Nomura et al, 1988, 1996), and in another the ethnic composition was not stated (Carter et al, 1990). Six studies had matched for years since blood sample was taken (Nomura et al, 1988, 1996; Carter et al, 1995; Gann et al, 1996; Guess et al, 1997; Vatten et al, 1997; Dorgan et al, 1998); three for time of day of venipuncture (Nomura et al, 1988, 1996; Barrett-Connor et al, 1990; Dorgan et al, 1998); two for previous history of prostatic surgery (Nomura et al, 1988, 1996; Gann et al, 1996), and two for smoking (Gann et al, 1996; Dorgan et al, 1998).

To present the results in a consistent format, the mean concentration of each hormone for cases and controls was extracted from the published data, and the ratio of the mean among cases to that of controls was calculated. Either serum or plasma concentrations were used, but considering their similarity, they shall be referred to as serum values. For each individual study, the standard errors of the means were used to calculate an approximate standard error of the log ratio. This was then used to obtain a 95% confidence interval (CI) for the log ratio which was exponentiated to give a corresponding CI for the ratio. Median values rather than means were published in three studies, and additional information was sought from the authors in order to obtain the means and standard errors for cases and controls.

In order to summarize the results, weighted averages of the study-specific log ratios were calculated, with weights determined by the inverse of the variance of each log ratio. The degree of heterogeneity in the log ratios between individual studies was assessed by $\chi^2$ tests.

The results are displayed separately for each hormone in figures 1 to 11 where each study-specific estimate of the ratio of the mean concentration in cases to controls is plotted as a square and its CI is denoted by the horizontal line crossing through it. The area of each square is inversely proportional to the variance of the log ratio and reflects the amount of statistical information available for that particular estimate. Where the CI extends beyond the scale of the plot it is indicated by an arrow. The summary estimate (the weighted average of the log ratios) is plotted as an open diamond, the horizontal tips of which represent the 95% CI.

*© 1999 Cancer Research Campaign* 

**1** Testosterone

| First author | Year | Cases/controls | ratio | 95% CI |
|---|---|---|---|---|
| Barrett-Connor | 1990 | 59/945 | 0.95 | (0.85–1.06) |
| Hsing | 1993 | 98/98 | 1.02 | (0.89–1.17) |
| Carter | 1995 | 16/16 | 0.83 | (0.62–1.11) |
| Gann | 1996 | 222/390 | 1.02 | (0.94–1.10) |
| Nomura | 1996 | 141/141 | 1.00 | (0.91–1.10) |
| Guess | 1997 | 106/106 | 0.99 | (0.90–1.09) |
| Vatten | 1997 | 59/180 | 0.97 | (0.88–1.07) |
| Dorgan | 1998 | 116/231 | 0.98 | (0.91–1.06) |
| All studies | | 817/2107 | 0.99 | (0.95–1.02) |

Test for heterogeneity $\chi^2_7 = 0.3$; $P > 0.1$, NS

**2** Non-SHBG bound testosterone

| First author | Year | Cases/controls | ratio | 95% CI |
|---|---|---|---|---|
| Nomura | 1996 | 105/105 | 1.04 | (0.90–1.19) |
| Guess | 1997 | 104/106 | 1.01 | (0.89–1.14) |
| Dorgan | 1998 | 116/231 | 1.03 | (0.95–1.11) |
| All studies | | 325/442 | 1.02 | (0.96–1.09) |

Test for heterogeneity $\chi^2_2 = 0.1$; $P > 0.1$, NS

**3** Dihydrotestosterone

| First author | Year | Cases/controls | ratio | 95% CI |
|---|---|---|---|---|
| Hsing | 1993 | 98/98 | 1.00 | (0.89–1.12) |
| Gann | 1996 | 222/390 | 0.97 | (0.88–1.07) |
| Nomura | 1996 | 141/141 | 1.03 | (0.94–1.14) |
| Vatten | 1997 | 59/180 | 0.98 | (0.88–1.10) |
| Dorgan | 1998 | 116/231 | 0.96 | (0.88–1.04) |
| All studies | | 636/1040 | 0.98 | (0.94–1.03) |

Test for heterogeneity $\chi^2_4 = 1.6$; $P > 0.1$, NS

**4** Androstanediol glucuronide

| First author | Year | Cases/controls | ratio | 95% CI |
|---|---|---|---|---|
| Gann | 1996 | 222/390 | 1.06 | (0.98–1.14) |
| Nomura | 1996 | 141/141 | 1.09 | (0.97–1.23) |
| Guess | 1997 | 106/106 | 1.02 | (0.91–1.16) |
| Vatten | 1997 | 59/180 | 1.02 | (0.90–1.17) |
| Dorgan | 1998 | 116/231 | 1.06 | (0.93–1.21) |
| All studies | | 644/1048 | 1.05 | (1.00–1.11) |

Test for heterogeneity $\chi^2_4 = 0.8$; $P > 0.1$, NS

**5** Androstenedione

| First author | Year | Cases/controls | ratio | 95% CI |
|---|---|---|---|---|
| Barrett-Connor | 1990 | 59/948 | 0.98 | (0.87–1.10) |
| Nomura | 1996 | 141/141 | 1.05 | (0.97–1.13) |
| Dorgan | 1998 | 116/231 | 0.99 | (0.93–1.06) |
| All studies | | 316/1320 | 1.01 | (0.97–1.06) |

Test for heterogeneity $\chi^2_1 = 1.4$; $P > 0.1$, NS

**6** Dehydroepiandrosterone sulphate

| First author | Year | Cases/controls | ratio | 95% CI |
|---|---|---|---|---|
| Comstock | 1993 | 81/81 | 0.88 | (0.72–1.09) |
| Dorgan | 1998 | 116/231 | 1.02 | (0.90–1.16) |
| All studies | | 197/312 | 0.98 | (0.88–1.10) |

Test for heterogeneity $\chi^2_1 = 1.3$; $P > 0.1$, NS

**Figures 1–6** Ratios of the mean hormone levels between cases and controls for testosterone, non-SHBG-bound testosterone, dihydrotestosterone, androstanediol glucuronide, androstenedione and dehydroepiandrosterone sulphate

### RESULTS

Figures 1–11 summarize the results of eight prospective studies which reported the serum concentration of testosterone (T), non-SHBG bound T, di-hydrotestosterone (DHT), androstanediol glucuronide (A-diol-g), androstenedione (A-dione), dehydroepiandrosterone sulphate (DHEAS), sex hormone binding globulin (SHBG), oestrone, oestradiol, luteinizing hormone (LH) and/or prolactin in men who subsequently developed prostate cancer in comparison to men who remained free of the disease. Most information was available for T with 817 cases. There was no evidence that the serum concentration of T was different between cases and controls, with a pooled ratio of 0.99 (95% CI 0.95–1.02) and there was no evidence of heterogeneity between the studies. The pooled estimates for the other hormones included data from between 114 and 644 cases and no significant differences in mean concentrations between prostate cases and controls were found. However, all five studies that measured A-diol-g reported a higher mean concentration among cases relative to controls which was reflected in the pooled ratio of 1.05 (95% CI 1.00–1.11). Thus, cases had, on average, a 5% higher mean serum concentration of A-diol-g than controls, based on 644 cases and 1048 controls.

### DISCUSSION

This quantitative review reveals no convincing evidence that serum levels of endogenous sex hormones, their precursor compounds and metabolites and related binding protein, differ between men who subsequently go on to develop prostate cancer and those who do not. The pooled estimate for A-diol-g does, however, suggest that prostate cancer cases had a 5% higher serum concentration relative to controls.

Differences in oestradiol levels of approximately 15% have been found to influence the risk of hormone-sensitive cancers, as found for breast cancer risk in post-menopausal women (Thomas et al, 1997). The finding that circulating serum levels of A-diol-g are 5% higher in prostate cancer cases than controls is consistent with the hypothesis that elevated hormonal activity is important in the progression of hormone-sensitive cancers. Circulating androgen levels are likely to be only weakly correlated with the hormonal environment within the prostate gland, therefore small differences in circulating serum levels of A-diol-g may reflect larger differences in androgen activity within the prostate gland that are of biological relevance. Elevated A-diol-g concentrations may reflect an increased transformation from T to DHT within prostatic tissue, resulting in overstimulation of cell growth and

© 1999 Cancer Research Campaign

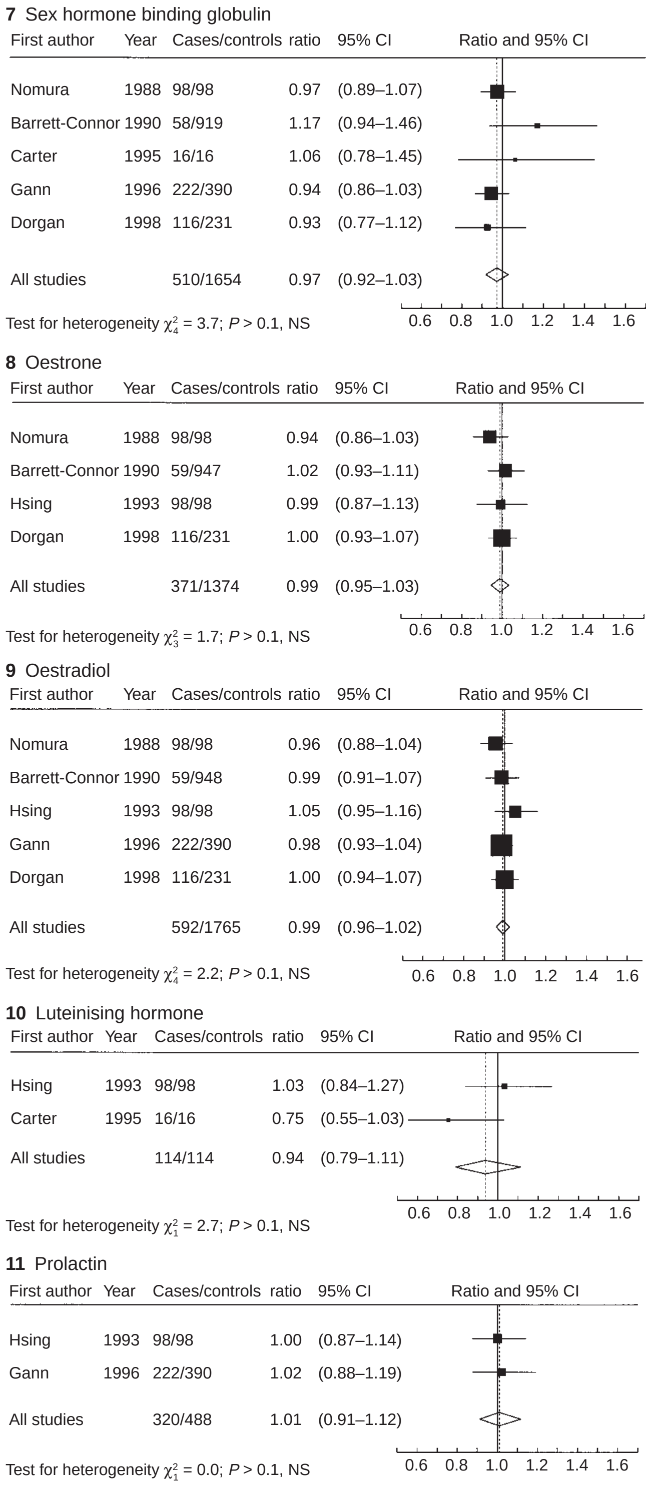

**Figures 7–11** Ratio of mean hormone levels between cases and controls for sex hormone binding globulin, oestrone, oestradiol, luteinising hormone and prolactin progression from subclinical tumour foci into a clinically-manifest form. Differences in serum A-diol-g concentrations have also been observed between racial groups with differential risks for prostate cancer, with higher levels being found among Caucasian and African-American men compared with Japanese men (Ross et al, 1992). These hormonal differences could be partly determined by dietary factors (Adlercreutz, 1990), although they may also have a strong genetic basis. For example, polymorphisms of the 5α-reductase(II) gene and the 3-α/β-hydroxysteroid dehydrogenase (II) gene have been found to be differentially distributed between populations in relation to prostate cancer risk (Reichardt et al, 1995; Devgan et al, 1997). These genes encode for enzymes involved in androgen metabolism within prostatic cells and allelic variants that alter enzymic activity could influence hormone metabolism and therefore may affect prostate cancer risk.

This is a systematic review of published data. We did not attempt to uncover unpublished observations or those published in other languages and it is possible that these may affect our pooled results. This review has presented results from each study in a consistent format, using the natural logarithm of the ratio of the mean concentrations of each hormone between cases and controls in preference to the differences in absolute concentrations. However, readers should refer to the original articles for details of the distribution of hormone values.

Little is known about the determinants of hormone levels and how they interact with each other to influence cell growth. As such, the measurements of T should be interpreted with care as T and SHBG are known to be strongly positively correlated via the feedback effect of free-T on the hypothalamic–pituitary–gonadal axis (Gann et al, 1996). Indeed, this study reported a much stronger association between T and prostate cancer risk once adjustment for SHBG had been made. As such, the lack of association found in this review may actually be confounded by the effect of SHBG and mask a stronger independent relationship between T and prostate cancer risk.

Although all studies included cases which were confirmed with histological examination or medical notes, only three studies analysed hormone levels according to whether cases were clinically overt cancer or were latent, subclinical prostate cancer diagnosed following transurethral resection for benign prostatic hyperplasia (Nomura et al, 1988, 1996; Hsing and Comstock, 1993; Dorgan et al, 1998). Similarly, the prevalence of indolent prostate tumours that existed amongst the control groups at the time of follow-up, was also unknown. This is a particular problem in prostate cancer epidemiology as prostatic carcinomas can be incidentally found at autopsy in up to 80% of men over the age of 80 (Breslow et al, 1977). Such misclassification will serve to dilute any association between hormone levels and cancer.

These results suggest that the differences, if any, in circulating hormone levels between prostate cancer cases and controls are small. However, this may, in part, be due to significant laboratory variation which will serve to dilute any association present. Furthermore, all studies, except Carter et al (1995), measured hormone levels in a single serum sample some years prior to diagnosis. This may lead to regression dilution bias towards the null due to random variation in hormone levels within individuals over time. Although a single measurement of circulating serum T and DHT concentrations is thought to reliably reflect mean annual androgen concentrations in middle-aged men (Vermeulen and Verdonck, 1992), little is known about the long-term reliability of a single serum measurement for other hormones. As such, the reported values of circulating concentrations of androgen metabolites and their related proteins should be interpreted with caution. Furthermore, it is difficult to determine to what extent a single measure reflects a lifetime index of hormone status or long-term exposure. Hormone status early on in life, such as during adolescence (Ross et al, 1986) or even as early as in utero (Henderson et

*© 1999 Cancer Research Campaign*

al, 1988), may be markedly different between high- and low-risk populations, and it may be that differences in circulating hormone levels at these critical times of development are more relevant to subsequent prostate cancer risk. It may therefore be more appropriate to collect several specimens from an individual over many years to achieve the necessary sensitivity to detect small differences in hormone levels.

This quantitative review suggests that there are no large differences between prostate cancer cases and controls in serum hormone concentrations measured in samples taken some years prior to diagnosis. Serum concentrations of A-diol-g were consistently slightly higher in men who subsequently went on to develop prostate cancer, and may reflect higher androgenic activity in these men.

### ACKNOWLEDGEMENTS

We would like to thank Dr Barrett-Connor, Ricki Bettencourt, Dr Guess and Dr Gann for providing data additional to those which had been published. This project was funded by the Imperial Cancer Research Fund.

### REFERENCES

Adlercreutz H (1990) Western diet and western diseases: some hormonal and biochemical mechanisms and associations. *Scand J Clin Lab Invest* **50**: 3–23

Barrett-Connor E, Garland C, McPhillips JB, Khaw KT and Wingard DL (1990) A prospective, population-based study of androstenedione, estrogens and prostatic cancer. *Cancer Res* **50**: 169–173

Breslow N, Chan CW, Dhom G, Drury RA, Franks LM, Gellei B, Lee Y, Lundberg S, Sparke B, Sternby NH and Tulinius H (1977) Latent carcinoma of the prostate at autopsy in seven areas. *Int J Cancer* **20**: 680–688

Carter HB, Pearson JD, Metter EJ, Chan DW, Andres R, Fozard JL, Rosner W and Walsh PC (1995) Longitudinal evaluation of serum androgen levels in men with and without prostate cancer. *Prostate* **27**: 25–31

Comstock GW, Gordon GB and Hsing AW (1993) The relationship of serum dehydroepiandrosterone and its sulfate to subsequent cancer of the prostate. *Cancer Epidemiol Biomark Prev* **2**: 219–221

Devgan SA, Henderson BE, Yu MC, Shi CY, Pike MC, Ross RK and Reichardt JKV (1997) Genetic variation of 3β-hydroxysteroid dehydrogenase type II in three racial/ethnic groups:implications for prostate cancer risk. *Prostate* **33**: 9–12

Dorgan JF, Albanes D, Virtamo J, Heinonen OP, Chandler DW, Galmarini M, McShane LM, Barrett MJ, Tangrea J and Taylor PR (1998) Relationships of serum androgens and estrogens to prostate cancer risk: results from a prospective study in Finland. *Cancer Epidemiol Biomark Prev* **7**: 1069–1074

Flanders WD (1986) Case-control studies of selected hormones and prostate cancer: an epidemiological review. *Alabama J Med Sci* **23**: 439–443

Gann PH, Hennekens CH, Ma J, Longcope C and Stampfer MJ (1996) Prospective study of sex hormone levels and risk of prostate cancer. *J Natl Cancer Inst* **88**: 1118–1126

Guess HA, Friedman GD, Sadler MC, Stanczyk FZ, Vogelman JH, Imperato-McGinley J, Lobo RA and Orentreich N (1997) 5α-reductase activity and prostate cancer: a case-control study using stored sera. *Cancer Epidemiol Biomark Prev* **6**: 21–24

Henderson BE, Bernstein L, Ross RK, Depue RH and Judd HL (1988) The early in utero estrogen and testosterone environment of blacks and whites: potential effects on male offspring. *Br J Cancer* **57**: 216–218

Hsing AW and Comstock GW (1993) Serological precursors of cancer: serum hormones and risk of subsequent prostate cancer. *Cancer Epidemiol Biomark Prev* **2**: 27–32

Montie JE and Pienta KJ (1994) Review of the role of androgenic hormones in the epidemiology of benign prostatic hyperplasia and prostate cancer. *Urology* **43**: 892–899

Nomura AMY, Heilbrun LK, Stemmermann GN and Judd HJ (1988) Prediagnostic serum hormones and the risk of prostate cancer. *Cancer Res* **48**: 3515–3517

Nomura AMY and Kolonel LN (1991) Prostate cancer: a current perspective. *Am J Epidemiol* **13**: 200–207

Nomura AMY, Stemmermann GN, Chyou PH, Henderson BE and Stanczyk FZ (1996) Serum androgens and prostate cancer. *Cancer Epidemiol Biomark Prev* **5**: 621–625

Parker SL, Tong T, Boldon S and Wingo PA (1996) Cancer statistics, 1996. *Cancer J Clin* **46**: 5–27

Parkin DM, Muir CS, Whelan SL, Gao Y, Ferlay J and Powell J (1992) *Cancer Incidence in Five Continents*, Vol VI. IARC Scientific Publication No. 129. International Agency for Research on Cancer: Lyon, France

Reichardt JKV, Makridakis N, Henderson BE, Yu MC, Pike MC and Ross RK (1995) Genetic variability of the human SRD5A2 gene: implications for prostate cancer risk. *Cancer Res* **55**: 3973–3975

Ross RK, Bernstein L, Judd H, Hanisch R, Pike MC and Henderson BE (1986) Serum testosterone levels in healthy young black and white men. *J Natl Cancer Inst* **76**: 45–48

Ross RK, Bernstein L, Lobo RA, Shimizu H, Stanczyk FZ, Pike MC and Henderson BE (1992) 5α-reductase activity and risk of prostate cancer among Japanese and US white and black males. *Lancet* **339**: 887–897

Shimizu H, Ross RK, Bernstein L, Yatani R, Henderson BE and Mack TM (1991) Cancers of the breast and prostate among Japanese and white immigrants to Los Angeles County. *Br J Cancer* **63**: 963–966

Thomas HV, Reeves GK and Key TJA (1997) Endogenous estrogen and postmenopausal breast cancer: a quantitative review. *Cancer Causes Control* **8**: 922–928

Vatten LJ, Ursin G, Ross RK, Stanczyk FZ, Lobo RA, Harvei S and Jellum E (1997) Androgens in serum and the risk of prostate cancer: a nested case-control study from the Janus serum bank in Norway. *Cancer Epidemiol Biomark Prev* **6**: 967–969

Vermeulen A and Verdonck G (1992) Representativeness of a single point plasma testosterone level for the long term hormonal milieu in men. *J Clin Endocrinol Metab* **74**: 939–942

*© 1999 Cancer Research Campaign*
